# pyKVFinder: an efficient and integrable Python package for biomolecular cavity detection and characterization in data science

**DOI:** 10.1186/s12859-021-04519-4

**Published:** 2021-12-20

**Authors:** João Victor da Silva Guerra, Helder Veras Ribeiro-Filho, Gabriel Ernesto Jara, Leandro Oliveira Bortot, José Geraldo de Carvalho Pereira, Paulo Sérgio Lopes-de-Oliveira

**Affiliations:** 1 Brazilian Center for Research in Energy and Materials (CNPEM), Brazilian Biosciences National Laboratory (LNBio), R. Giuseppe Máximo Scolfaro, 10000 - Bosque das Palmeiras, Campinas, SP 13083-100 Brazil; 2 Graduate Program in Pharmaceutical Sciences, Faculty of Pharmaceutical Sciences, University of Campinas, Campinas, SP Brazil

**Keywords:** Cavity detection, Cavity characterization, NumPy, Python, Data structure, Data science, Automated pipelines, Molecular dynamics

## Abstract

**Background:**

Biomolecular interactions that modulate biological processes occur mainly in cavities throughout the surface of biomolecular structures. In the data science era, structural biology has benefited from the increasing availability of biostructural data due to advances in structural determination and computational methods. In this scenario, data-intensive cavity analysis demands efficient scripting routines built on easily manipulated data structures. To fulfill this need, we developed pyKVFinder, a Python package to detect and characterize cavities in biomolecular structures for data science and automated pipelines.

**Results:**

pyKVFinder efficiently detects cavities in biomolecular structures and computes their volume, area, depth and hydropathy, storing these cavity properties in NumPy arrays. Benefited from Python ecosystem interoperability and data structures, pyKVFinder can be integrated with third-party scientific packages and libraries for mathematical calculations, machine learning and 3D visualization in automated workflows. As proof of pyKVFinder’s capabilities, we successfully identified and compared ADRP substrate-binding site of SARS-CoV-2 and a set of homologous proteins with pyKVFinder, showing its integrability with data science packages such as matplotlib, NGL Viewer, SciPy and Jupyter notebook.

**Conclusions:**

We introduce an efficient, highly versatile and easily integrable software for detecting and characterizing biomolecular cavities in data science applications and automated protocols. pyKVFinder facilitates biostructural data analysis with scripting routines in the Python ecosystem and can be building blocks for data science and drug design applications.

**Supplementary Information:**

The online version contains supplementary material available at 10.1186/s12859-021-04519-4.

## Background

At present, we are going through the era of data science and the computational and structural biology fields have hugely benefited from the growing availability of biostructural data, as pointed out by [1]: “Structural biology meets data science”. Advances in X-ray crystallography and electron microscopy techniques have expanded the determination of novel structures [2]. In the meantime, advances in computational resources have driven the use of in silico methods to simulate the dynamics of biomolecules and carried out the implementation of artificial intelligence to model biomolecular structures [3]. Together, all of this structural data provides fertile ground for data interpretation through data science or automated analysis frameworks, but data-intensive analysis asks for efficient and integrable scripting routines with an easily manipulated data structure.

In this scenario, we developed pyKVFinder, an open-source python package for cavity detection and characterization abstracted into multidimensional arrays. In proteins or other macromolecules, solvent-exposed clefts or buried cavities play a key role in ligand binding, which can ultimately regulate biological function of the macromolecule [4]. For this reason, the identification and characterization of ligand-binding sites are the basis of rational structure-based drug discovery and design [5, 6]. pyKVFinder adopts the original geometrical grid-and-sphere-based detection method as implemented in KVFinder [7], which has been improved in the latest parallel version, parKVFinder [8]. Detected cavities in parKVFinder, as in many other well-known programs, such as CavVis [9], fpocket [10], GHECOM [11], ConCavity [12], MSpocket [13] and POVME 3.0 [14], are human-readable and easily displayed in molecular visualization programs, but are not properly structured to be directly scripted into automated pipelines or data science frameworks. Programming languages are constantly evolving in the data science field, with Python being one of the most popular in the community [15]. However, only a few initiatives have been launched to make cavity detection programs easier to integrate into data science protocols. For instance, Cambridge Crystallographic Data Centre has licensed commercial software suites that use a Python API to integrate its structural database with workflows and third-party applications [16], and one of its API modules is aimed at detecting ligand-binding cavities using the LIGSITE algorithm [17]. As an open-source initiative, Biobb_vs is a Python package designed to detect and analyze cavities in automated workflows. Biobb_vs is part of BioExcel Building Blocks [18] and uses fpocket to detect cavities in virtual screening pipelines. In this case, despite the interoperability achieved, the workflows depend on handling data files generated by fpocket.

To fulfill this need, pyKVFinder is wrapped into Python and, using Python’s well-established data structure (e. g., NumPy array), benefits from the language ecosystem interoperability. pyKVFinder can be integrated with third-party scientific packages and libraries for mathematical calculations, statistical analysis, and tridimensional visualization. Moreover, users can explore the functionality of pyKVFinder step-by-step using interactive interfaces, such as IPython/Jupyter notebooks. As mentioned above, one of pyKVFinder’s main contributions in data science workflows is to translate the detected cavities from tridimensional coordinates of cavity points to NumPy arrays, a data structure that allows for a wide diversity of scientific computation and efficient storage and access to N-dimensional arrays (ndarrays), also called tensors [19]. Ndarrays also provide efficient ways of handling data for mathematical operations and are the popular choice of input data type for machine learning Python libraries such as scikit-learn [20].

Besides conventional cavity properties such as volume and area, which are stored as Python dictionaries, pyKVFinder computes cavity depth and hydropathy. Similar to cavity points, these spatial and physicochemical properties are stored as Python ndarrays and can be visualized using Python molecular visualization widgets. Thus, pyKVFinder provides a versatile way to detect and characterize biomolecular cavities and integrate this information into data science or automated workflows.

## Implementation

Python-C parallel KVFinder (pyKVFinder) applies a Simplified Wrapper and Interface Generator (SWIG; http://www.swig.org/) to extend grid operations written in C to Python, a high-level programming language. pyKVFinder can be easily installed with the pip package management system. In pyKVFinder, the target biomolecule is inserted into a regular 3D grid, which is stored as an ndarray, considering the van der Waals radii of the atoms. To detect cavities, pyKVFinder uses a dual-probe algorithm that scans the biomolecular structure, as described in [7, 8]. In summary, a small probe, dubbed Probe In, and a larger probe, dubbed Probe Out, translate over the grid points, defining two molecular surfaces with different accessibility. Biomolecular cavities are defined as the non-overlapped regions between the molecular surfaces. On each detected cavity, pyKVFinder may perform spatial, depth, hydropathy and constitutional characterizations. In this workflow, C routines undergo multithreaded parallelization to speed up cavity detection and characterization, with the OpenMP API creating a user-defined number of parallel threads, where grid chunks are distributed among these threads to perform independent operations.

### Python package

**Fig. 1:**
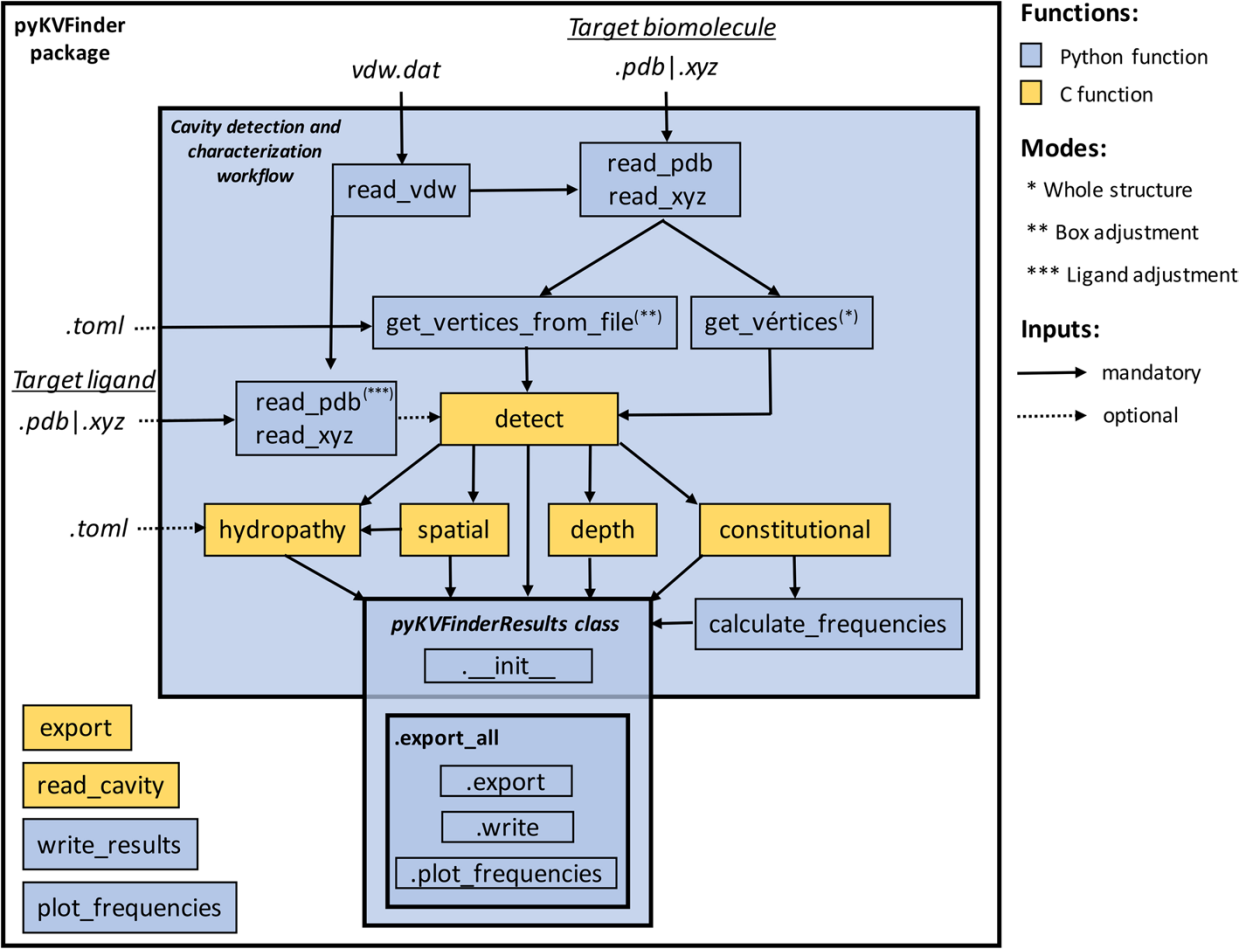
Diagram of cavity detection and characterization workflow using pyKVFinder package. The flowchart illustrates function calls and their dependencies for performing cavity detection and characterization with pyKVFinder package

pyKVFinder can be imported as a package in the Python environment and users can decide to run the full cavity detection and characterization workflow through the *run_workflow* function or run pyKVFinder functions in a stepwise fashion. At the latter, users can integrate pyKVFinder functions into third-party Python packages and benefit from interactive IPython/Jupyter notebooks. By running pyKVFinder in Python environment, users can visualize the detected cavities through the Python NGL Viewer widget [21]. Still, the full workflow was also coded as a command-line interface. Either way, users have access to a full set of customizable parameters to detect and characterize biomolecular cavities. A schematic diagram of cavity detection and characterization workflow is described in Fig. 1.

In the cavity detection and characterization workflow, *read_vdw* function reads a customizable van der Waals (vdW) radii file (*.dat* extension; default *vdw.dat*) into a nested Python dictionary, which is first indexed by the three-letter residue code (e. g., ALA, ARG, ASH, etc.), and then indexed by the atom name (e. g., C, N, O, CA, etc.) to access its respective radius value. The vdW radii file defines the radius values for each atom by residue and when not defined, uses a generic value based on the atom type. The built-in file (*vdw.dat*) applies the vdW radii of the Amber ff99 force field [22]. Afterwards, *read_pdb* or *read_xyz* function gets the vdW dictionary and reads a target PDB or XYZ file (*.pdb* or.*xyz* extension), respectively. The atomic data is stored in an ndarray with residue number, chain identifier, residue name, atom name, xyz coordinates and radius per atom.

**Fig. 2:**
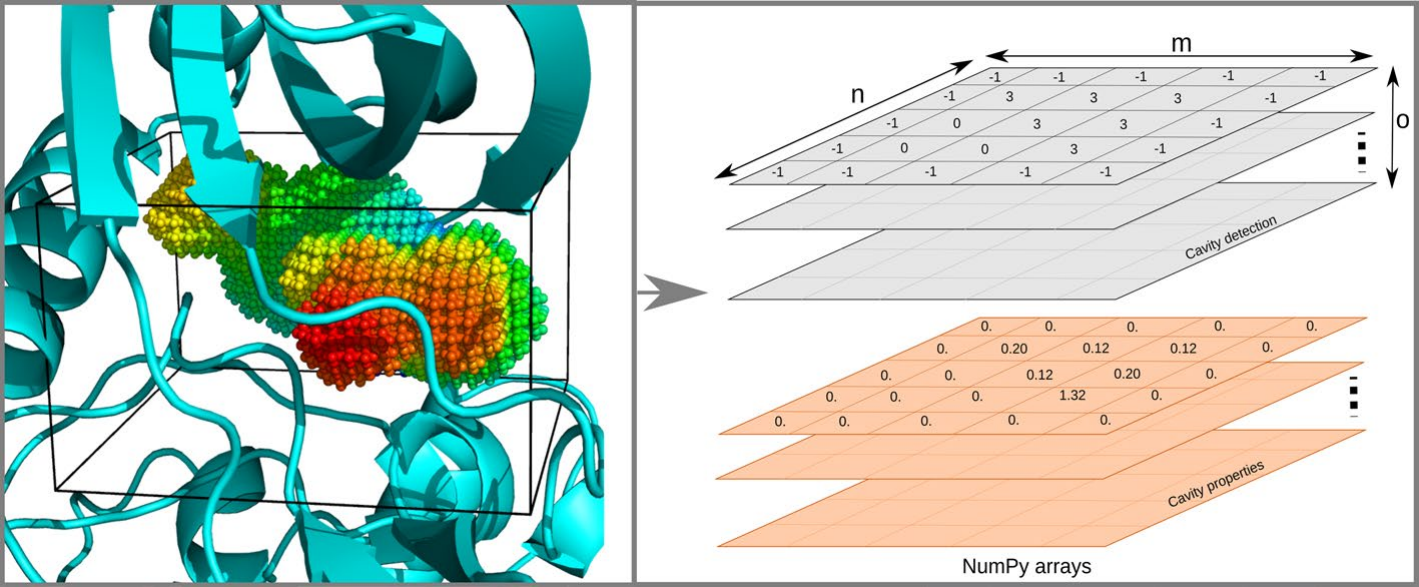
Representative view of detected cavities in the pyKVFinder data structure. Based on a 3D grid (left figure), pyKVFinder detects cavities in biomolecules and returns an ndarray with dimensions (m, n, o) (right figure). Each ndarray element corresponds to cavity space (> 1), empty space (1), biomolecule space (0) or bulk space (-1). Cavity properties such as depth and hydropathy are also stored in the same data structure, with ndarray elements corresponding to the property value

To perform the cavity detection on the whole structure, the 3D grid is defined based on the target biomolecule coordinates and the user-defined parameters: grid spacing and Probe Out diameter. Thus, the grid coordinates are extracted from the atomic data ndarray, using *get_vertices* function that defines the grid origin and XYZ axis. The first vertex (p1) is the minimum xyz coordinates of the atomic data. The second (p2), third (p3) and fourth (p4) vertices represent the maximum coordinate along the X, Y and Z axes, respectively. Given these four vertices, the original coordinate system is transformed in the *detect* function, using translation and rotation, into a new coordinate system with P1 as its origin. Also, for internal calculations, the sum of the grid spacing and the Probe Out size is padded in each direction of the 3D grid. With user-defined parameters and atomic coordinates, the *detect* function creates and fills the 3D grids with Probe In and Probe Out probes, and compares these grids to define the biomolecular cavities, which are returned in an ndarray. In this ndarray (Fig. 2), each element corresponds to cavity space (> 1), empty space (1), biomolecule space (0) or bulk space (− 1).

Instead of performing the cavity detection on the whole structure, the search space can be set to a custom search box, called box adjustment mode. This mode is defined based on a TOML-formatted configuration file (*.toml* extension; Additional file 1: Fig. A1), which can explicitly define the vertices coordinates or pass a list of residues of the target biomolecule to be used as reference instead of the whole structure. The box can also be drawn using parKVFinder PyMOL plugin and its coordinates can be extracted from parKVFinder parameters file. Thus, the *get_vertices_from_file* function loads a box configuration file and atomic data from *read_pdb* or *read_xyz* function, which returns the grid coordinates (p1, p2, p3 and p4) and selects the atoms inside the custom 3D grid with their respective atomic data. Hence, these parameters are passed to *detect* function, together with *box_adjustment* flag, to perform cavity detection on a custom 3D grid.

Either way, whole structure mode or box adjustment mode, another space segmentation method, called ligand adjustment mode, can be applied to constrain the search space around a target ligand, defined by a PDB or XYZ file (*.pdb* or.*xyz* extension). For this mode, *read_pdb* or *read_xyz* function gets the vdW dictionary and reads a ligand PDB or XYZ file and returns the ligand atomic coordinates with their respective radius that must be passed to *detect* function to further restrict the search space within a radius of the target ligand.

### Cavity characterization

With the ndarray of detected cavities, pyKVFinder package may perform four characterization procedures, i.e., spatial, constitutional, depth and hydropathy characterizations. The spatial characterization, using *spatial* function, defines the surface points in an ndarray, and estimates the volume and area of the detected cavities, following the methodology proposed in [8]. The constitutional characterization, using *constitutional* function, defines the interface residues that surround the detected cavities, checking if the atoms of the residues are within a radius, which is the sum of the Probe In size and the atom radius. Alternatively, the *constitutional* function can ignore backbone contacts by enabling *ignore_backbone* flag. This function stores the interface residues in a Python dictionary. Using the interface residues, the *calculate_frequencies* function calculates the occurrence of each residue and classes of residues per cavity. The classes of amino acid residues [23] are aliphatic apolar (R1), aromatic (R2), polar uncharged (R3), negatively charged (R4), positively charged (R5) and non-standard (RX) (Additional file 1: Table A1).

The depth characterization identifies the degree of burial of the binding site. This characterization applies *depth* function to identify the cavity-bulk boundary by applying a spatial filter, which defines a boundary point as cavity points where at least one direct neighbor is a bulk point, marking it with the negative of the cavity integer (Additional file 1: Fig. A2). Subsequently, the depth of each cavity point is heuristically estimated by the shortest Euclidean distance between the cavity point and its respective boundary points (Eq. 1). Each cavity point is distributed among the user-defined number of threads to perform this chunk of distance calculations (Additional file 1: Fig. A2). With the depth of all cavity points calculated and stored as an ndarray (Fig. 2), the maximum and average depths are calculated for each detected cavity and returned as Python dictionaries.

1 $$\widehat{{D_{i} }} = \mathop {min}\limits_{j} \left[ {d\left( {p_{i} ,p_{j} } \right)} \right] = \mathop {min}\limits_{j} \left[ {\sqrt {\left( {p_{{i_{x} }} - p_{{j_{x} }} } \right)^{2} + \left( {p_{{i_{y} }} - p_{{j_{v} }} } \right)^{2} + \left( {p_{{i_{z} }} - p_{{j_{z} }} } \right)^{2} } } \right],$$

where $\widehat{{D_{i} }}$ is the depth of the cavity point i, $d\left( {p_{i} ,p_{j} } \right)$ is the Euclidean distance between points i and j, $p_{i}$ is the x, y, z coordinates of a cavity point i, $p_{j}$ is the x, y, z coordinates of a cavity-bulk boundary point j.

The hydropathy characterization extracts the water attractiveness of the interface residues surrounding the binding site. This characterization uses *hydropathy* function at surface points detected in spatial characterization to map a target hydrophobicity scale on them. Firstly, a customizable TOML-formatted hydrophobicity scale file (*.toml* extension) is loaded into a Python dictionary, which is indexed by the three-letter residue code (e. g., ALA, ARG, ASH, etc.) to access the respective hydrophobicity value. With the dictionary loaded, the function identifies the nearest interface residues for each surface point and projects the residue’s hydrophobicity value onto them (Fig. 2). Alternatively, backbone contacts may be ignored by enabling the *ignore_backbone* flag. Finally, with the hydrophobicity mapped on the surface and returned as an ndarray, the average hydropathy is calculated for each detected cavity and stored in a Python dictionary. The hydrophobicity scale file defines the scale name and hydrophobicity value for each residue and, when not defined, assigns zero to the missing residues (Additional file 1: Fig. A3). The package contains six built-in hydrophobicity scales: Eisenberg and Weiss [24], Hessa and Heijne [25], Kyte and Doolittle [26], Moon and Fleming [27], Wimley and White [28] and Zhao and London [29]. Benefiting from the Python environment, users can test different scales without having to perform the cavity detection step every time. A pre-released version of hydropathy characterization has been successfully applied to compare cavities of alphavirus-related proteins [30].

**Fig. 3:**
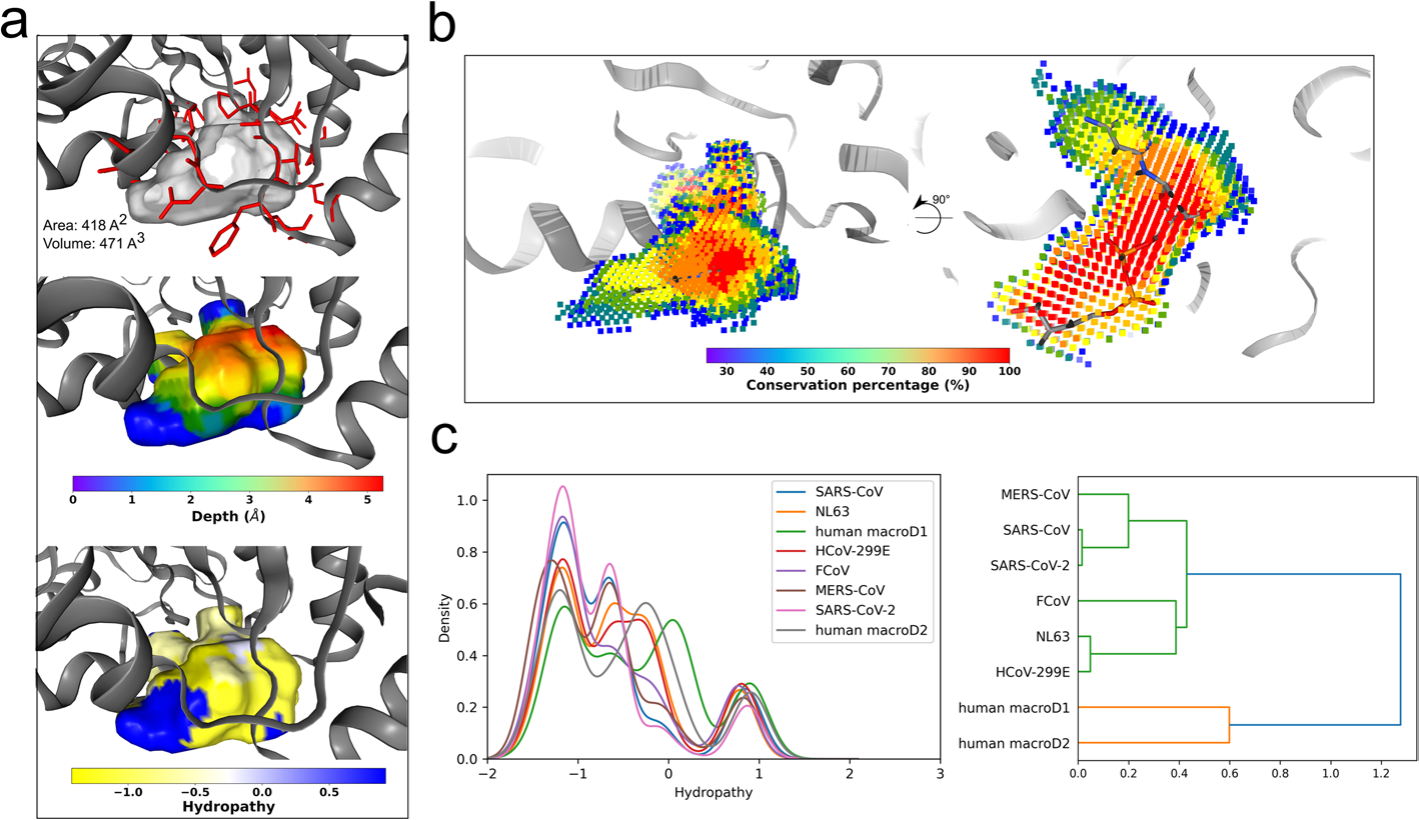
Detection and characterization of ADRP substrate-binding cavity of SARS-CoV-2 and its comparison to related coronaviruses and human macroD1 and macroD2 proteins. **a** Three different characterizations of the apo ADRP substrate-binding cavity of SARS-CoV-2 (PDB ID: 6WEN) using pyKVFinder. The upper panel shows the detected cavity represented as gray surface and residues surrounding it as red sticks. The cavity area and volume are displayed. The middle panel presents the same cavity colored by depth, while the bottom panel shows the cavity colored by hydropathy using Eisenberg and Weiss scale. **b** Conservation analysis of the ADP-ribose binding site in ADRP domain of SARS-CoV-2 (PDB ID: 6WEN, chain A), SARS-CoV (PDB ID: 2ACF, chain B), MERS-CoV (PDB ID: 5HIH, chain A), NL63 (PDB ID: 2VRI, chain A), HCoV-229E (PDB ID: 3EJG, chain A), FCoV (PDB ID: 3ETI, chain B) and human macrodomain proteins macroD1 (PDB ID: 2X47, chain A) and macroD2 (PDB ID: 6Y73, chain D) from human. These protein domains were selected using Dali and choosing homologs in apo form. The structures were realigned using MUSTANG algorithm [39] from YASARA program [40]. The figure presents cavity points that were detected in at least two structures and the points are colored by conservation percentage. **c** Hydropathy profile of the same compared cavities collected from pyKVFinder ndarrays. **d** Hierarchical clustering dendrogram of the frequency of residues surrounding the compared cavities. The correlation metric was used to assess the similarity and the complete method was chosen as linkage method. All the images and graphics were created inside a Jupyter notebook. To create images of tridimensional structures, we used NGL Viewer tool and to plot graphics, we used matplotlib library

The cavity detection and characterization objects, from *detect*, *spatial*, *constitutional*, *calculate_frequencies*, *depth*, *hydropathy* functions, can be stored into a *pyKVFinderResults* class that accumulates them in its attributes. As this data structure is filled, these attributes can be exported to files through *export*, *write* and *plot_frequencies* methods. The *export* method writes cavities together with their surface points to a PDB-formatted file with depth values in the B-factor column, while surface points with hydropathy values in the B-factor column are written to another PDB-formatted file. The *write* method saves file paths, volume, area, interface residues and their frequencies, maximum and average depth, and average hydropathy in a TOML-formatted file. The *plot_frequencies* method plots bar charts of frequencies per cavity in a PDF file. These three methods are also wrapped in *export_all* method. Although we presented the full workflow, all functions explained in this section can be applied independently in a step-by-step manner. In this scenario, the *export*, *write* and *plot_frequencies* methods have their counterparts in the *export*, *write_results* and *plot_frequencies* functions of pyKVFinder package, respectively. Additionally, the *read_cavity* function reads a cavity file (*.pdb* extension), written by pyKVFinder, parKVFinder or KVFinder, and a target PDB or XYZ file (*.pdb* or*.xyz* extension), and returns an ndarray with each element corresponding to the cavity space (> 1), biomolecule space (0), or bulk or empty space (− 1), similar to the output of the *detect* function. In this way, it allows to recharacterize a previously detected cavity or characterize a cavity with manually trimmed points.

## Results and discussion

### Usage example

To demonstrate the use of pyKVFinder and how it benefits from the Python ecosystem, we identified the substrate-binding pocket of the ADP-ribose phosphatase (ADRP) domain of SARS-CoV-2 nsp3 protein in the apo form (PDB ID: 6WEN). Still under investigation to determine its exact functions in coronavirus life cycle, the ADRP domain recognizes ADP-ribose 1″ phosphate [31, 32] and seems to have an important role in virulence and innate immunity regulation to infection [33–35]. In this regard, recent efforts have been made to characterize ADP-ribose substrate-binding pocket and evaluate this site as a putative antiviral drug target [36, 37].

### Visualizing detected cavities with NGL Viewer in Jupyter notebook

pyKVFinder successfully detected the ADRP substrate-binding cavity and determined traditional cavity properties such as volume, area and residues surrounding the ADP-ribose cavity (Fig. 3a, upper panel). For instance, we used pyKVFinder *calculate_frequencies* and *plot_frequencies* functions to determine the composition of the type of residues surrounding the cavity and plotted this composition as a bar chart (Additional file 1: Fig. A4). In pyKVFinder, this step is performed using matplotlib library [38], but users are free to analyze data and present results on their favorite graphing library. As observed in Fig. 3a, the ADP-ribose site forms a cleft sandwiched between ADRP α-helices and the main contacts involve residues from coil regions, which could possibly explain the pocket plasticity upon substrate binding [31, 32]. These results were visualized using NGL Viewer on a Jupyter notebook; alternatively, users can use another molecular visualization tool for notebooks or load results into the parKVFinder PyMOL Plugin [8].

**Fig. 4:**
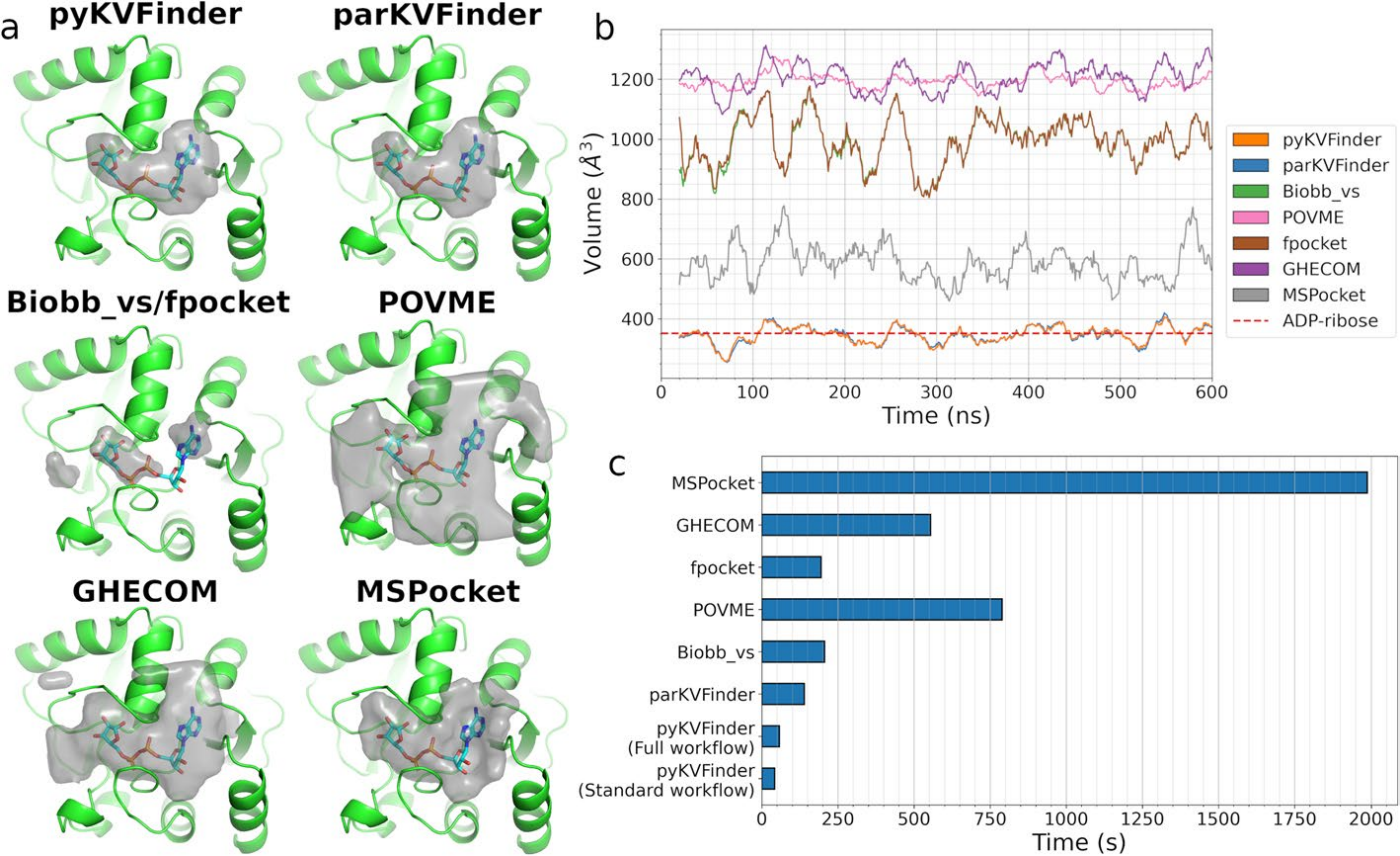
Performance evaluation of the benchmarking methods for detecting the ADRP substrate-binding site. **a** The structures of the protein (green cartoon) at frame 30 (smallest RMSD compared to the crystallographic structure) of the ADRP domain trajectory with the corresponding cavities detected (gray surfaces) by each benchmarking method. **b** The total volume of the cavities detected in the ADRP substrate-binding site along the 600 ns simulation. The total volume is averaged in a window of 20 frames. The red dashed line indicates the molecular surface volume of the ADP-ribose molecule that originally occupied the ADRP substrate-binding site in the crystallographic structure (PDB ID: 6W02, chain B). **c** Elapsed time to detect and characterize ADRP substrate-binding site. The standard workflow of pyKVFinder, as in parKVFinder, detects cavities and applies spatial and constitutional characterizations. The full workflow of pyKVFinder comprises standard workflow with depth and hydropathy characterizations

### Characterizing hydropathy and depth of cavities

We also inspected the ADRP substrate-binding cavity through other two points of view: depth and hydropathy. Those physicochemical descriptions are usually essential for drug development [36]. In apo form, despite being solvent-exposed, the cavity has some internal components (red color) that can reach a more central portion of the ADRP β-sheet (Fig. 3a, middle panel). The hydropathy analysis shows that the cavity core is most hydrophobic (yellow color), with some polar residues on the edges (blue color) that may contribute to the design of more specific ligands. Since pyKVFinder stores the properties to be colored in cavities in the B-factor column of a PDB-formatted file, users can easily change the style and color scheme in most of molecular visualization programs.

### Using NumPy operations to present conservation and matplotlib library to plot hydropathy distribution of cavities

In addition to SARS-CoV-2, the ADRP domain is also present in other related coronaviruses and has the macroD1 and macroD2 as homologous in humans. For this reason, we used pyKVFinder to detect the ADRP substrate-binding site in aligned ADRP domains from different species and compare their properties. Firstly, we applied arithmetic operations on the ndarrays of the detected cavities to determine the cavity conservation among the species. As observed in Fig. 3b, the ADRP cavity has a core (red points) very conserved in the analyzed species which is occupied by the diphosphate and ribose of ADP and the second ribose bound to ADP in the ADRP substrate-bound form. In turn, adenosine occupies a less conserved cavity region, which may indicate that the structure of this site in some species changes to accommodate ADP-ribose substrate. To compare the cavity hydropathy across species, we plotted a hydropathy distribution from the hydropathy ndarray using the matplotlib library [38] (Fig. 3c, left graph). The distribution clearly shows the hydrophobic characteristic of the pocket that is mostly shared between ADRP substrate-binding pockets of coronaviruses. Interestingly, the human macroD1 and macroD2 seem to shift the distribution to a less hydrophobic profile. This finding should be better evaluated, as the differences between these homologous domains that share the same substrate can contribute to the design of specific ligands for viral ADRP domains.

### Hierarchical clustering of cavity residues using SciPy package

Finally, since pyKVFinder uses native Python dictionaries to store the residues surrounding the detected cavity, we can easily tabulate the residue frequency. With this information, we performed a hierarchical clustering, an unsupervised machine learning algorithm, using the SciPy package [41], and represented clusters arrangement as a dendrogram (Fig. 3c, right graph). The ADRP cavity of SARS-CoV-2 grouped with that of SARS-CoV, demonstrating the high identity between these betacoronaviruses. Close to them, we can observe another betacoronavirus, MERS-CoV. On the other hand, the alphacoronaviruses NL63 and HCoV-229E and the feline FCoV are grouped together. Further away from the coronaviruses’ domains are the two human macrodomain proteins, macroD1 and D2. Despite the cavity of ADRP or macro D1/D2 sharing the same substrate, ADP-ribose, these results show that the profile of the residues surrounding these cavities follows evolutionary traces.

### Benchmarking

In addition to identifying and characterizing the ADRP substrate-binding site of SARS-CoV-2 and a set of homologous proteins, we simulated ADRP domain of SARS-CoV-2 (PDB ID: 6W02, chain B) without its ligand, ADP-ribose, for 600 ns, extracting a frame at regular intervals of 1 ns (Additional file 1). Thus, we used pyKVFinder with its box adjustment mode to detect and estimate the volume of the ADP-ribose binding site throughout 600 frames of the ADRP domain’s trajectory. This analysis was repeated with other well-known software: POVME [14], Biobb_vs [18], MSPocket [13], GHECOM [11], fpocket [10] and parKVFinder [8]. Biobb_vs, as mentioned in the Background section, is a Python package that allow scripting, while POVME, MSpocket, GHECOM, fpocket, GHECOM and parKVFinder are command-line interfaces. A detailed description of software parameters and versions is in Additional file 1.

All these methods successfully detected the pocket of the ADRP substrate-binding site, in which the shape and volume vary slightly during the molecular dynamics simulation (Fig. 4). The shape of the detected cavities defined by pyKVFinder and parKVFinder finely adjust to the original ligand in the binding site, as well as MSPocket (Fig. 4a) Besides that, the volume calculated by pyKVFinder (346.8 ± 78.7 Å^3^) and parKVFinder (346.5 ± 79.3 Å^3^) is closely related to the volume of ADP-ribose (351.1 Å^3^; molecular surface volume estimated by YASARA program [40]), the ligand that originally occupied the binding site in the crystallographic structure used in the molecular dynamics simulations (Fig. 4b). Nevertheless, the differences in the shape and volume of detected cavities derive from the methodology employed (e.g., Voronoi tessellation, alpha spheres, and grid-and-sphere), the cavity-bulk boundary definition, and the ability to segment the space. For instance, pyKVFinder, parKVFinder and POVME can segment the search space, which trims points outside this custom space, while the other methods only explore the whole structure, which includes neighboring regions at the binding site.

Besides being able to accurately detect biomolecular cavities, current software must also perform fast detection and characterizations. Thus, we also evaluated the elapsed time to execute these benchmarking methods (Fig. 4c). pyKVFinder outperformed all analyzed methods. Even when applying the newly available characterization, depth and hydropathy, pyKVFinder's elapsed time only increased 36%, still outperforming other benchmarking methods. Further, compared to its counterpart, parKVFinder, pyKVFinder was 3.3 times faster in detecting ADRP binding site. The main reason for the performance gain is the additional possibility to parallelize routines, i. e., the insertion of atoms in the 3D grid in *detect* function, based on ndarrays. Hence, experienced users requiring scripting routines are encouraged to use pyKVFinder due to its improved performance, while newcomers should prioritize parKVFinder due to its simplicity of installation and execution. Further, the scalability of pyKVFinder, upon increasing number of threads, follows the same behavior presented by parKVFinder [8].

Despite all methods characterizing volume, each method has its own set of characterizations to be performed on the detected cavities. However, the cavities data structure is only accessible inside the Python ecosystem in pyKVFinder, which provides ndarrays and Python dictionaries. The ndarrays stores cavity points, surface points, hydropathy for each surface point and depth for each cavity point, while Python dictionaries stores volume, area, average hydropathy, maximum depth and average depth, and interface residues and their frequencies per detected cavity. Thus, users may develop new characterizations and/or analysis pipelines with these data structures.

### Future development

pyKVFinder will undergo continuous improvements and updates, according to its applications by the scientific community. In the future, pipelines will be implemented in molecular dynamics and machine learning, along with new features that are valuable to ligand-binding site characterization. Additionally, pyKVFinder aims to offload its routines to the GPU for performance enhancement in data-intensive applications.

## Conclusion

pyKVFinder provides an efficient and integrable Python package for cavity detection and characterization in biomolecular structures for data science and automated pipelines. In addition to fast, accurate and efficient cavity detection and characterization, pyKVFinder stores spatial and physicochemical properties in Python ndarrays, that ease scripting and data analysis. Further, pyKVFinder performance was benchmarked against well-known geometrical methods for cavity detection and characterization. Finally, we have successfully shown an application of pyKVFinder integration with matplotlib, NGL Viewer, SciPy and Jupyter notebook, that compared the ADRP substrate-binding site of SARS-CoV-2 in homologous proteins.

## Availability and requirements

- **Project name:** pyKVFinder
- **Project home page:** https://github.com/LBC-LNBio/pyKVFinder
- **Operating system(s):** any supporting Python >  = 3.7 (tested on Linux and macOS)
- **Programming language:** Python, C
- **Other requirements:** swig >  = 4.0.1, toml >  = 0.10.2, numpy >  = 1.20.3, matplotlib >  = 3.3.3
- **License:** GNU General Public License v3.0
- **Any restrictions to use by non-academics:** None.

## Supplementary Information

**Additional file 1.** The Additional file 1 contains **Table A1**, **Figures A1**, **A2**, **A3** and **A4**, and a detailed description of the molecular dynamics simulation of ADRP domain of SARS-CoV-2 and the benchmarking procedure. **Table A1** reports the classes of amino acid residues. **Figure A1** shows examples of box configuration files. **Figure A2** shows the methodology of depth characterization. **Figure A3** shows the methodology of hydropathy characterization. **Figure A4** shows a bar chart of residues frequencies.

## Data Availability

pyKVFinder source code, documentation and tutorials are available in the Python Package Index (PyPI) repository, https://pypi.org/project/pyKVFinder, and the GitHub repository, https://github.com/LBC-LNBio/pyKVFinder. Documentation and tutorials are available at pyKVFinder webpage, https://lbc-lnbio.github.io/pyKVFinder.

## Abbreviations

ADRP: ADP-ribose phosphatase
CoV: Coronavirus
MERS: Middle East Respiratory Syndrome
ndarrays: N-dimensional arrays
PDB: Protein Data Bank
R1: Aliphatic apolar
R2: Aromatic
R3: Polar uncharged
R4: Negatively charged
R5: Positively charged
RX: Non-standard
SARS: Severe Acute Respiratory Syndrome
SWIG: Simplified Wrapper and Interface Generator
TOML: Tom's Obvious, Minimal Language
vdW: Van der Waals
