# Supplementary material for: pyKVFinder: an efficient and integrable Python package for biomolecular cavity detection and characterization in data science
Source: BMC Bioinformatics. 2021 Dec 20;22:607. doi: 10.1186/s12859-021-04519-4 (PMC8685811; doi:10.1186/s12859-021-04519-4)
Supplement: Supplementary file 1 — Additional file 1. The Additional file 1 contains Table A1, Figures A1, A2, A3 and A4, and a detailed description of the molecular dynamics simulation of ADRP domain of SARS-CoV-2 and the benchmarking procedure. Table A1 reports the classes of amino acid residues. Figure A1 shows examples of box configuration files. Figure A2 shows the methodology of depth characterization. Figure A3 shows the methodology of hydropathy characterization. Figure A4 shows a bar chart of residues frequencies. [file 12859_2021_4519_MOESM1_ESM.pdf]

Additional File 1: Supplementary information

Table A1: Classes of amino acid residues. R1: Aliphatic apolar; R2: Aromatic; R3: Polar uncharged; R4: Negatively charged; R5: Positively charged; RX: Non-standard.

Amino acid residue		Classification [1]
**Alanine (Ala)**	R1	Aliphatic apolar
**Glycine (Gly)**		Aliphatic apolar
**Isoleucine (Ile)**		Aliphatic apolar
**Leucine (Leu)**		Aliphatic apolar
**Proline (Pro)**		Aliphatic apolar
**Valine (Val)**		Aliphatic apolar
**Phenylalanine (Phe)**	R2	Aromatic
**Tryptophan (Trp)**		Aromatic
**Tyrosine (Tyr)**		Aromatic
**Asparagine (Asn)**	R3	Polar uncharged
**Cysteine (Cys)**		Polar uncharged
**Glutamine (Gln)**		Polar uncharged
**Methionine (Met)**		Polar uncharged
**Serine (Ser)**		Polar uncharged
**Threonine (Thr)**		Polar uncharged
**Aspartic Acid (Asp)**	R4	Negatively charged
**Glutamic Acid (Glu)**		Negatively charged
**Arginine (Arg)**	R5	Positively charged
**Histidine (His)**		Positively charged
**Lysine (Lys)**		Positively charged

Figure A1: Examples of box configuration file based on (a) grid coordinates and (b) a list of residues and a padding.

a
```
[box]
# px = [x, y, z]
p1 = [0.0, 0.0, 0.0]
p2 = [1.0, 0.0, 0.0]
p3 = [0.0, 1.0, 0.0]
p4 = [0.0, 0.0, 1.0]
```

b
```
[box]
residues = [ ["resname", "chain",], ["resname", "chain",], ]
padding =  3.5
```

Figure A2: Methodology of depth characterization. (a) The spatial filter evaluates if the cavity point (black) is a direct neighbor (red) of a bulk point to assign it as a boundary point. (b) The distance between the cavity point (black) and the boundary points (red) are calculated, and the shortest distance (blue line) is the depth of it.

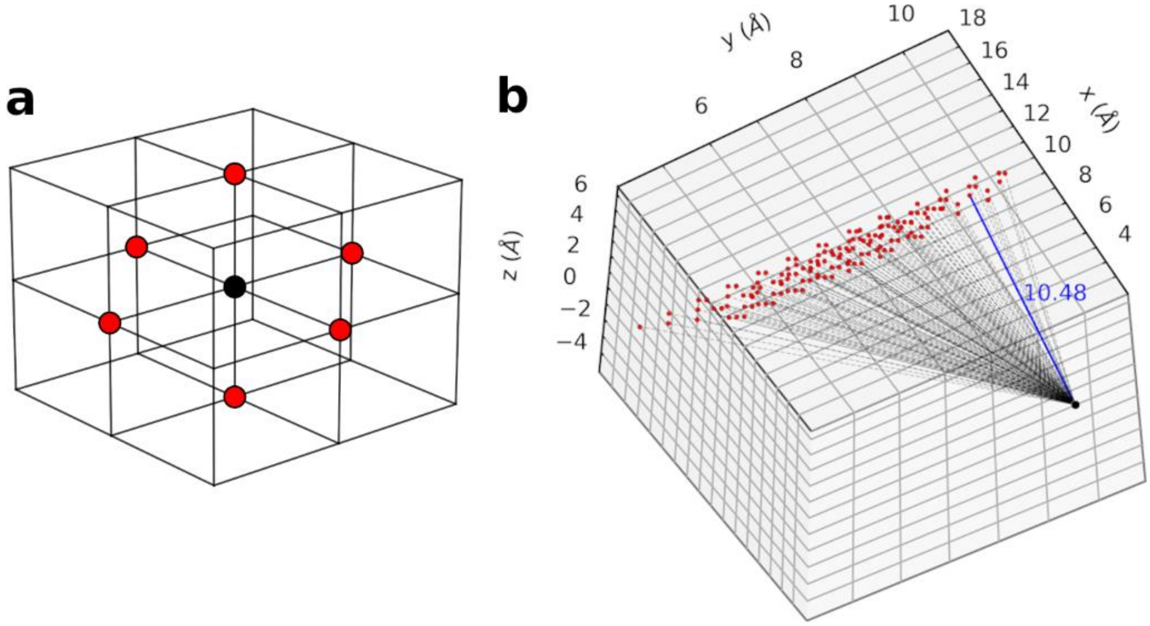

Figure A3: Methodology of hydropathy characterization. (a) Example of TOML-formatted hydrophobicity scale file. (b) The distance between the surface point (black) and the atoms (carbon: green; oxygen: red; nitrogen: blue; sulfur: yellow) of the residues (methionine: left; valine: right) are calculated, and the residue of the atom with shortest distance (red line) maps its hydrophobicity value on it.

a

```
[EisenbergWeiss]
ALA = -0.64
ARG = 2.6
ASN = 0.8
ASP = 0.92
CYS = -0.3
GLN = 0.87
GLU = 0.76
GLY = -0.49
HIS = 0.41
ILE = -1.42
LEU = -1.09
LYS = 1.54
MET = -0.66
PHE = -1.22
PRO = -0.12
SER = 0.18
THR = 0.05
TRP = -0.83
TYR = -0.27
VAL = -1.11
```

b

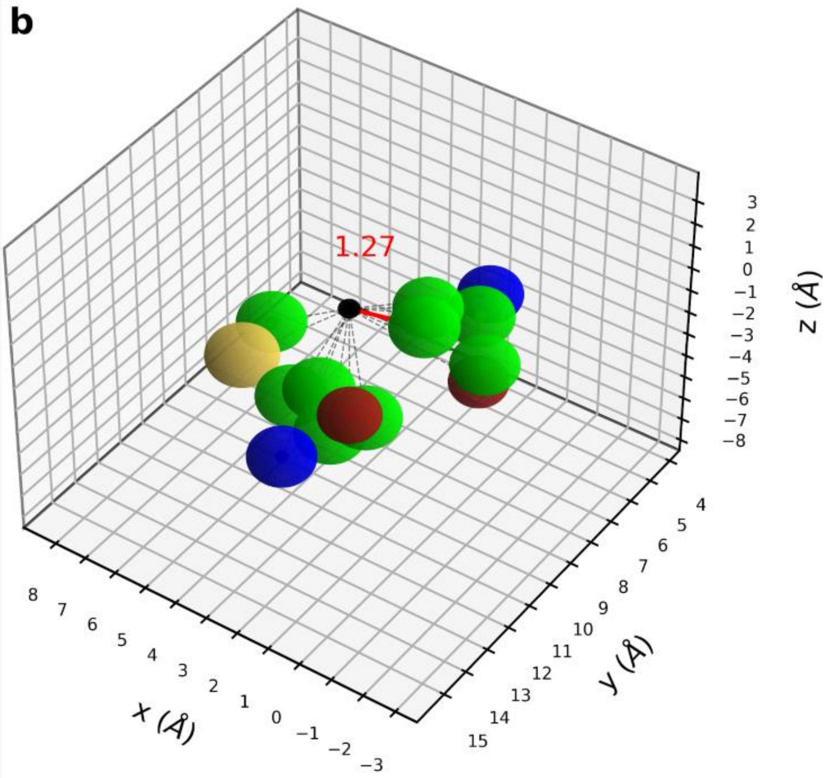

Figure A4: Bar chart of residues frequencies. Left: Frequency of types of residues. Right: Frequency of classes of residues.

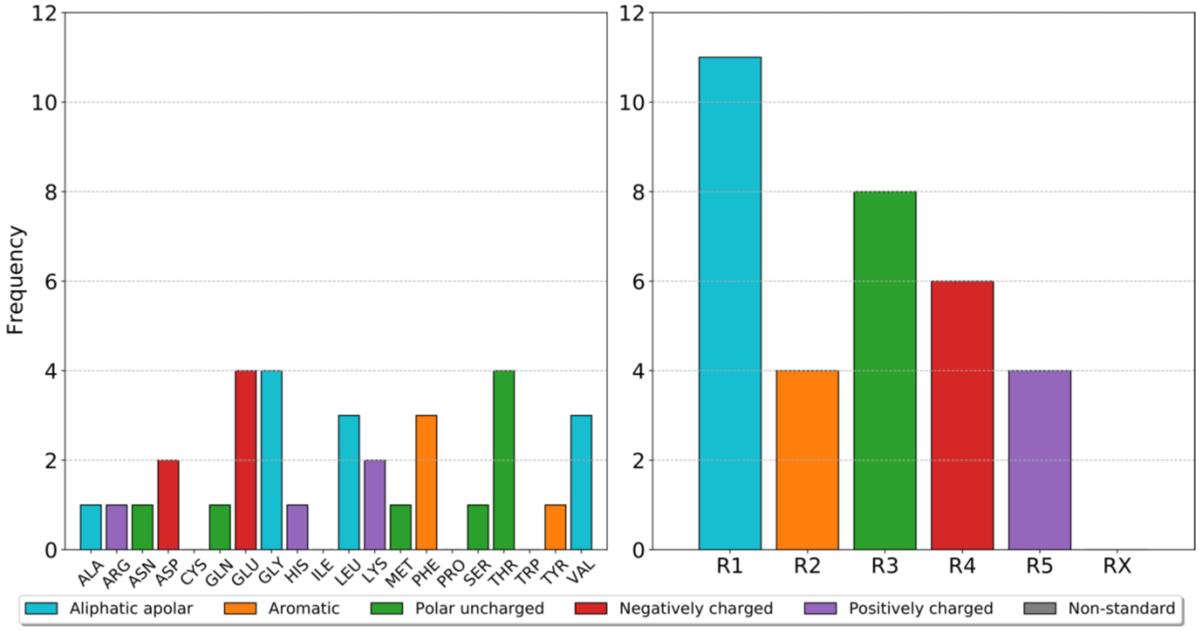

Molecular dynamics simulation of ADRP domain of SARS-CoV-2

Molecular dynamics simulations were performed with AMBER 18 GPU code [2–4] using the ff14SB force field [5] with the TIP3P water model [6]. All covalent bonds involving hydrogen atoms were constrained using SHAKE algorithm [7] to allow the time step of 2 fs. The solvated system was submitted to 2500 energy minimization steps of steepest descent algorithm, followed by 2500 steps of conjugate gradient algorithm. Then, an equilibration was performed with an NVT simulation, followed by and NPT equilibration run, with a simulation time of 0.2 ns and 0.5 ns, respectively. For these simulations, a restrain was applied on all the protein atom positions with a force constant of 10 kcal mol^{-1} $Å^{-2}$. The production run of 650 ns was performed, and the last 600 ns were employed for analysis.

All the simulations were run at 298.15 K and 1 bar. The temperature control was done using a Langevin thermostat [8] with a collision frequency of 5 ps^{-1}, while the pressure control was performed with a Berendsen barostat [9] and a relaxation time of 1 ps^{-1}, respectively. Particle Mesh Ewald method [10] were applied for calculating long-electrostatic with a cut-off of 9 Å. The initial structure was the ADRP domain without the ADP-ribose that is present in the crystallographic structure (PDB ID: 6W02, chain B) [11]. The molecular dynamics simulations were performed on a desktop computer with a 16-core 4.7 GHz AMD Ryzen 9 3950X processor, 128 GB RAM and an NVIDIA GeForce RTX 2080 SUPER GPU, running Ubuntu 20.04 operating system.

Benchmarking procedure

For the analysis, the molecular dynamics' trajectory was aligned using MDLovoFit [12]. The RMSD of the 70% less mobile alpha-carbons was used as alignment criteria. A total of 600 frames were extracted at regular intervals of 1 ns from the trajectory. Each frame was independently analyzed with the benchmarking methods using the parameters described.

For all methods, the default parameters were changed only when necessary to ensure accurate representation of the ADRP substrate binding site throughout the trajectory. pyKVFinder (v.0.2.5) and parKVFinder (v.1.1.3; https://github.com/LBC-LNBio/parKVFinder) detected cavities with Grid Spacing of 0.8 Å, Probe Out of 6 Å and Volume Cutoff of 25 $Å^3$, segmenting the search space with an optimized search box (box adjustment mode) that comprises the ADP-ribose (APR) present in binding site of the crystallographic structure (PDB ID: 6W02, chain B). POVME 3.0 (https://github.com/POVME/POVME) was run with an InclusionBox with the same coordinates of the one used in pyKVFinder and contiguous points criteria of 3. Biobb_vs (v.3.7.0; https://github.com/bioexcel/biobb_vs), fpocket (v3.0; https://github.com/Discngine/fpocket), MSPocket (v1.1; https://projects.biotec.tu-dresden.de/MSPocket/) and GHECOM (https://pdbj.org/ghecom/) were run with default parameters. All parameters not mentioned were kept at their default values. These calculations were performed on a desktop computer with a 6-core 3.4 GHz AMD Ryzen 5 2600 processor and 32 GB RAM, running Ubuntu 20.04.2 LTS operating system. Furthermore, all methods were executed with the maximum number of threads available. The parallel software (pyKVFinder, parKVFinder and POVME) executed the analysis with 12 threads, using the maximum number of threads of the processor, while software that do not use parallel procedures (Biobb_vs, fpocket, MSPocket and GHECOM) were executed with a single thread.

The ADPR substrate binding site is split in sub-pockets by the movement of side-chains, e. g. phenylalanine-132 and phenylalanine-156. Based on this, to properly select

cavities to correctly describe the binding site, we selected all sub-pockets that had at least 50% of its composition inside a box, comprising the ADP-ribose (APR) present in binding site of the crystallographic structure, similarly to what was performed in pyKVFinder. This procedure was applied to the results of each frame of all benchmarking methods, except for pyKVFinder, parKVFinder and POVME because this analysis is already present in their own subroutines.

References

1. Nelson DL, Cox MM. Lehninger Principles of Biochemistry, Fourth Edition. Fourth Edition. 2004.

2. Salomon-Ferrer R, Götz AW, Poole D, Le Grand S, Walker RC. Routine Microsecond Molecular Dynamics Simulations with AMBER on GPUs. 2. Explicit Solvent Particle Mesh Ewald. J Chem Theory Comput. 2013;9:3878–88.

3. Götz AW, Williamson MJ, Xu D, Poole D, Le Grand S, Walker RC. Routine Microsecond Molecular Dynamics Simulations with AMBER on GPUs. 1. Generalized Born. J Chem Theory Comput. 2012;8:1542–55.

4. Le Grand S, Götz AW, Walker RC. SPFP: Speed without compromise—A mixed precision model for GPU accelerated molecular dynamics simulations. Comput Phys Commun. 2013;184:374–80.

5. Maier JA, Martinez C, Kasavajhala K, Wickstrom L, Hauser KE, Simmerling C. ff14SB: Improving the Accuracy of Protein Side Chain and Backbone Parameters from ff99SB. J Chem Theory Comput. 2015;11:3696–713.

6. Jorgensen WL, Chandrasekhar J, Madura JD, Impey RW, Klein ML. Comparison of simple potential functions for simulating liquid water. J Chem Phys. 1983;79:926–35.

7. Ryckaert J-P, Ciccotti G, Berendsen HJ. Numerical integration of the cartesian equations of motion of a system with constraints: molecular dynamics of n-alkanes. J Comput Phys. 1977;23:327–41.

8. Loncharich RJ, Brooks BR, Pastor RW. Langevin dynamics of peptides: The frictional dependence of isomerization rates ofN-acetylalanyl-N?-methylamide. Biopolymers. 1992;32:523–35.

9. Berendsen HJC, Postma JPM, van Gunsteren WF, DiNola A, Haak JR. Molecular dynamics with coupling to an external bath. J Chem Phys. 1984;81:3684–90.

10. Darden T, York D, Pedersen L. Particle mesh Ewald: An *N* ·log(*N*) method for Ewald sums in large systems. J Chem Phys. 1993;98:10089–92.

11. Michalska K, Kim Y, Jedrzejczak R, Maltseva NI, Stols L, Endres M, et al. Crystal structures of SARS-CoV-2 ADP-ribose phosphatase: from the apo form to ligand complexes. IUCrJ. 2020;7.

12. Martínez L. Automatic Identification of Mobile and Rigid Substructures in Molecular Dynamics Simulations and Fractional Structural Fluctuation Analysis. PLoS One. 2015;10:e0119264.
